# BRAF V600E mutational load as a prognosis biomarker in malignant melanoma

**DOI:** 10.1371/journal.pone.0230136

**Published:** 2020-03-13

**Authors:** Arrate Sevilla, M. Celia Morales, Pilar A. Ezkurra, Javier Rasero, Verónica Velasco, Goikoane Cancho-Galan, Ana Sánchez-Diez, Karmele Mujika, Cristina Penas, Isabel Smith, Aintzane Asumendi, Jesús M. Cortés, Maria Dolores Boyano, Santos Alonso

**Affiliations:** 1 Department of Genetics, Physical Anthropology and Animal Physiology, Faculty of Science and Technology, UPV/EHU, Leioa, Spain; 2 Department of Cell Biology and Histology, Faculty of Medicine and Nursing, UPV/EHU, Leioa, Spain; 3 Biocruces-Bizkaia Health Research Institute, Barakaldo, Spain; 4 Department of Pathology, Cruces University Hospital, Barakaldo, Spain; 5 Department of Pathology, Basurto University Hospital, Bilbao, Spain; 6 Department of Dermatology, Basurto University Hospital, Bilbao, Spain; 7 Department of Oncology, Onkologikoa Hospital, Donostia, Spain; 8 Biodonostia Health Research Institute, Donostia-San Sebastián, Spain; 9 Department of Zoology and Animal Cell Biology, Faculty of Science and Technology, UPV/EHU, Leioa, Spain; 10 Biocruces-Bizkaia Health Research Institute, Barakaldo, Spain; 11 Ikerbasque: The Basque Foundation for Science, Bilbao, Spain; Rutgers University, UNITED STATES

## Abstract

Analyzing the mutational load of driver mutations in melanoma could provide valuable information regarding its progression. We aimed at analyzing the heterogeneity of mutational load of BRAF V600E in biopsies of melanoma patients of different stages, and investigating its potential as a prognosis factor. Mutational load of BRAF V600E was analyzed by digital PCR in 78 biopsies of melanoma patients of different stages and 10 nevi. The BRAF V600E load was compared among biopsies of different stages. Results showed a great variability in the load of V600E (0%-81%). Interestingly, we observed a significant difference in the load of V600E between the early and late melanoma stages, in the sense of an inverse correlation between BRAF V600E mutational load and melanoma progression. In addition, a machine learning approach showed that the mutational load of BRAF V600E could be a good predictor of metastasis in stage II patients. Our results suggest that BRAF V600E is a promising biomarker of prognosis in stage II patients.

## Introduction

*BRAF* mutations are considered to be one of the earliest events in melanoma development [1]. The most common somatic mutation in *BRAF* is a V600E, accounting for 70% to 88% of all *BRAF* mutations [2]. V600E mutation is clinically relevant, because based on its presence, many patients receive targeted therapy, although paradoxically, BRAF V600E has been reported to be more frequent in benign (≈80%) than in dysplastic nevi (≈60%) or melanoma (≈40%-45%) [3, 4]. Thus, the detection of the BRAF V600E mutation in melanoma samples is used to select patients who should respond to *BRAF* inhibitors (like vemurafenib or dabrafenib), although unfortunately, most metastatic patients with initial tumor response develop resistance [5].

Different techniques are routinely used to determine *BRAF* status in clinical samples, the most widely used being the Cobas® 4800 BRAF V600 Mutation Test (Roche Molecular Diagnostics), based on a polymerase chain reaction (PCR). However, this test determines *qualitatively* the presence or absence of the mutation. In this sense, tumor heterogeneity can affect the sensitivity for somatic mutation detection, which may lead to false negatives [6].

Actually, malignant melanoma is a highly heterogeneous neoplasm, composed of subpopulations of tumor cells with distinct phenotypes [7], in which different subpopulations of the tumor may have different behavior and different response to treatments. In this sense, previous studies have reported that a high mutational load of BRAF V600E is associated with a better response to BRAF V600E inhibitors in stage III and IV patients [8].

Based on this evidence, it seems clear that a *quantitative* assessment of mutations would be more reliable and useful than a *qualitative* assessment. In this regard, digital PCR (dPCR) is an analytical technique for absolute quantitation of nucleic acid samples based on PCR amplification of single template molecules. dPCR works by partitioning a sample of DNA into thousands of individual, parallel PCRs. Following PCR analysis, the fraction of negative reactions is used to generate an absolute count of the number of target molecules in the sample, without the need for standards or endogenous controls. This method provides thus a sensitive and precise quantification of the load of particular mutations in tumor samples.

Therefore, we aimed at analyzing the heterogeneity in the mutational load of BRAF V600E in biopsies of melanoma patients of different stages at diagnosis, in order to investigate if the mutational load of BRAF V600E could serve as a useful prognosis factor.

## Materials & methods

### Ethics statement

The study protocol conformed to the tenets of the Declaration of Helsinki (Version Brazil 2013) and was approved by the Euskadi Ethics Committee (PI+CES BIOEF 2016–06). All patients (including those with nevi) gave written informed consent to participate in the study and to use their biopsies as material for research.

### Patients and samples

The study focused on 78 patients, all of them with histologically confirmed malignant melanoma: 41 women and 37 men; mean age 61 years (range 24–85: see Table 1). Patients were untreated, other than primary surgery. Disease stages were classified according to the AJCC (American Joint Committee on Cancer). A group of 10 nevi was also included in the study.

**Table 1:** Clinical characteristics of melanoma patients (n = 78).

	N
**Sex**	
Women	41
Men	37
**Age at diagnosis**	Range 24–85
**AJCC Stage at diagnosis**	
In situ	3
IA	7
IB	17
IIA	14
IIB	12
IIC	12
IIIA	2
IIIB	5
IIIC	2
IV	4
**Disease evolution**	
Disease free	43
Metastasis	35
**Localization**	
Head and Neck	14
Trunk	25
Upper extremity	6
Lower extremity	24
Hands and foot	6
Others	3
**Breslow Thickness (mm)**	
<1	17
1–4	35
>4	26

The melanoma patients at stage II were divided into two groups according to disease prognosis: patients with good prognosis (without metastasis in the time considered, median follow-up of 4 years) and metastatic patients (patients who developed metastasis after surgery, median, 1.4 years). Of the 38 stage II patients, 21 (55.26%) developed metastasis during follow-up.

### Immunohistochemistry (IHC)

The formalin fixed paraffin embedded (FFPE) biopsy sections (3 μm thick) were placed on 3-aminopropyletxylene-covered slides. Subsequently, sections were stained with mouse monoclonal antibody against BRAF V600E (1/100 dilution; clone VE1) following the Ventana Medical Systems' protocol and as previously described [9]. Briefly, staining was performed on a Ventana BenchMark Ultra (Ventana Medical Systems, Inc., Tucson, AZ, USA). The staining protocol included the use of Cell Conditioning 1 for 64 minutes; pre-peroxidase inhibition with 3% hydrogen peroxide for 10 minutes at 37°C and primary antibody incubation for 70 minutes. Amplification kit was applied for 4 minutes at 37°C to increase the signal intensity. The OptiView DAB IHC Detection kit was used to detect BRAF V600E protein expression. Tissues were counterstained with hematoxylin for 16 minutes and Bluing Reagent for 4 minutes. Then, stained sections were scanned by NanoZoomer S210 Digital slide scanner (Hamamatsu Photonics, Japan). The scoring of VE1 antibody staining was performed by visual inspection of two independent researchers. The scores range from 0 to 3 (0 for negative staining and 1–3 for different levels of positive staining).

### Digital PCR (dPCR)

DNA was isolated from 78 paraffin‐embedded skin biopsies from different melanoma stages, as well as from 10 nevi, using HigherPurity^TM^ FFPE DNA Isolation Kit (Canvax Biotech). To detect and quantify the V600E mutation, digital PCR was performed with QuantStudio 3D Digital PCR System (Applied Biosystems), in combination with Taqman dPCR Liquid Biopsy Assays (Hs000000004_rm and Hs000000003_rm). An evaluation of the technique was done on 10 commercial melanoma cell lines (S1 Table).

Data analysis was performed with the AnalysisSuite Software v3.1.4 (Applied Biosystems). Tumoral areas were measured using ImageJ software in stained hematoxylin-eosin scanned images. The areas occupied by stroma cells were excluded and the rate of melanocytes or melanoma area with respect to total area of the biopsy was applied to correct mutational load (S2 Text and S1 Fig).

QuantStudio 3D Digital PCR System was also used to determine the copy number of *BRAF* in some samples with BRAF V600E mutational load higher than 60%. We used the *BRAF* TaqMan Copy Number Assay (Hs04935060_cn) and TERT TaqMan^™^ Copy Number Reference Assay as a reference. Genomic copy number of BRAF was determined as the ratio between BRAF and TERT, multiplied by 2: $\frac{BRAF\;copies/ul}{RNaseP\;copies/ul}\;x\;2$.

### Statistical analysis and classification

Fisher’s exact test and Mann–Whitney U-test were used to determine the level of statistical significance between groups by means of the SPSS statistical software. Concordance between dPCR and IHC results was determined by Spearman’s *ρ* test.

Initial predictive modelling based on logistic regression, was performed using the 'caret' package for R. Then, a more standardized machine learning analysis was performed using 'scikit -learn'. We applied a machine learning Decision Tree classifier, with a Leave-One-Out cross-validation test to separate a metastatic response from a non-metastatic response.

For a power analysis of the effect size, we calculated the Cohen’s effect size.

## Results and discussion

**Fig 1:**
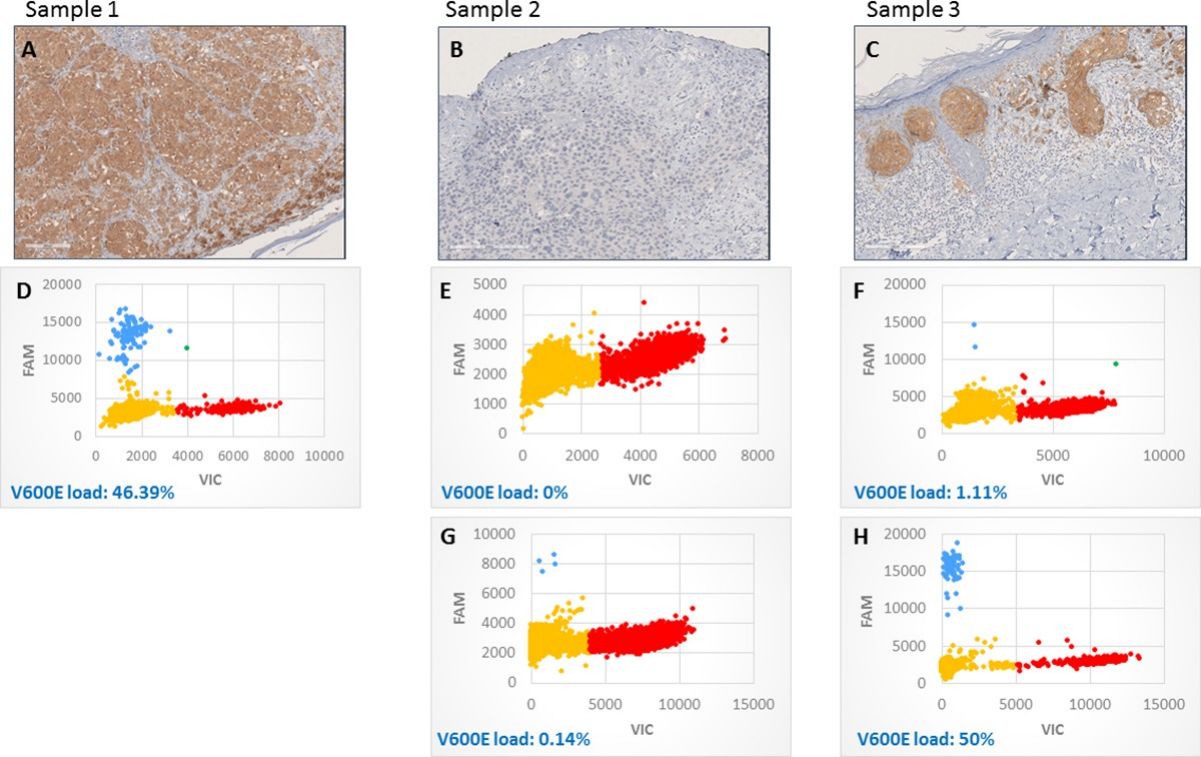
Immunohistochemistry (IHC) and digital PCR (dPCR) results from 3 samples. A-C) Immunohistochemical staining of sections of paraffin-embedded biopsies for BRAF V600E (VE1 antibody) contrasted with hematoxylin-eosin staining. D-H) dPCR results for BRAF V600E mutation. D-F) for c.1799T>A mutation and G-H) c.1799_1800TG>AA mutation. Every dot represents a well in the chip: In blue, wells containing BRAF V600E alleles; in red wells containing wild-type BRAF; in green, wells containing both V600E and wild-type forms, and in yellow, wells with no DNA molecules. Sample 1: IHC: V600E BRAF positive and dPCR: high mutational load for c.1799T>A. Sample 2: IHC: V600E BRAF negative. dPCR: negative for c.1799T>A and low mutational load for c.1799_1800TG>AA mutation. Sample 3: IHC: V600E BRAF positive. dPCR: Really low mutational load for c.1799T>A, but high for c.1799_1800TG>AA mutation.

We have analyzed the mutational load of BRAF V600E in 78 paraffin‐embedded skin biopsies from different melanoma stages, based on AJCC classification, as well as from 10 nevi. QuantStudio 3D Digital PCR System was used in combination with Taqman technology to detect and quantify the BRAF V600E mutation. To remove the confounding contribution of surrounding, healthy tissue, the BRAF V600E mutational load corresponding to the tumor tissue was obtained by correcting the obtained value by the proportion of tumor area in each biopsy based on immunohistochemistry (IHC) images obtained with the BRAF V600E antibody VE1 (Fig 1) (see Material and Methods).

Results show a great variability in the biopsies regarding their BRAF V600E mutational load, ranging from 0% to 81% (median 1.27%). Initially, we expected only values of mutational load between 0 and 50%, based on the assumption that the V600E mutation would be the result of a single, mutational event (i.e. only one mutant chromosome per diploid cell). To test whether the values of V600E mutational load > = 60% were the result of amplification, as suggested by Willmore-Payne et *al*. [10], we analyzed the copy number of *BRAF* in those 3 samples, all of which showed 3 or 4 copies of *BRAF*. Therefore our results confirm that in those samples with load of V600E > = 60% this is due to an amplification of *BRAF*.

Initially, the concordance between dPCR and IHC was relatively high, and significant (Spearman’s *ρ* = 0.652, p <0.01). The value of Spearman’s *ρ* can be explained by a few samples, which did not perfectly match: 17 samples were classified as negative by IHC and positive by dPCR. This discordance could be due to the higher precision of dPCR, as it is able to quantify rare mutants at a load of ≤0.1%. On the other hand, there were 4 samples which were clearly positive with IHC but negative or with low mutational load with dPCR. This discrepancy can be explained as the result of synonymous codons for V600E [11, 12]. To test if the 4 discrepant samples described above contained the V600E2 mutation (c.1799_1800TG>AA) instead of the canonical c.1799T>A, we performed dPCR with Taqman probes targeting c.1799_1800TG>AA. The results showed that all of the four samples had that mutation, one of them in a high percentage (about 50%). Based on these results, we also analyzed this mutation in the rest of the samples that had been classified as negative with the assay for c.1799T>A. Many of them presented the mutation, but the load was < 5% in all the cases (Fig 1). This was in accordance with other studies that show that the most common mutation is c.1799T>A [12].

Besides, we decided to analyze the mutational load of V600E in 10 nevi, because it is described that a great percentage of nevi present that mutation [13]. We also observed a great variability among nevi: 4 of them had a high mutational load (>35%), 2 had intermediate (5–10%) and 4 had very low mutational load (<2%) or the mutation was not present. Our results show that nevi can also be heterogeneous regarding BRAF V600E mutational load, in contrast with the assumption of a clonal origin of BRAF V600E mutation in melanocytic nevi proposed by Yeh et *al*. [1], and in accordance with the polyclonal nature proposed by Lin et *al*. [14].

In order to provide more information about the reproducibility of our method, we performed a replication of 17 samples. We isolated DNA from the same biopsies, but from different tissue slides of the paraffined block. The results showed a good correlation with the previous experiment, with a Pearson's r of 0.96 (p<0.01).

Taking all the above into account, we compared the V600E mutational load among different melanoma stages. We observed that most biopsies of early stages (0, IA, IB) have high mutational load of BRAF V600E, while in most late stages (II, III, IV) biopsies the mutational load is lower. Setting a threshold on 5% for the V600E mutational load, a significant difference was observed between early and late stages (Fisher’s exact test, one-tailed: p = 0.00487).

Among the samples analyzed, 3 were "*in situ*" melanomas (Table 1). Because *in situ* melanomas rarely progress into metastasis; and at the same time, nevi can be also misclassified as *in situ* melanomas, we removed these 3 samples and repeated the analysis. In this case, group differences were further increased (Fisher’s exact test, one-tailed: p = 0.00247). Mann-Whitney U-test also showed that there was a significant difference between early and late stages (p = 0.025). Altogether, these results suggest that there is an inverse correlation for BRAF V600E mutational load and melanoma progression.

Next, we focused on stage II biopsies, due to the abundance of samples in our study and the great variability of this stage regarding evolution to metastasis. While in general few patients with *in situ* and stage I melanomas develop metastasis, many stage II patients do develop metastasis. Among our patients with stage II melanomas (n = 38), more than a half (n = 21) developed metastasis. Consequently, we wondered if the mutational load of BRAF V600E could help in the prognosis of stage II patients.

Comparison of the mutational load of biopsies from stage II patients who developed metastasis after surgery for primary tumors, with that from patients who remained disease-free, showed that there is a statistically significant inverse correlation between BRAF V600E mutational load and melanoma metastasis risk in stage II biopsies (Mann–Whitney U-test: p = 0.018). We also compared both groups (metastatic and non-metastatic) vs percentage of BRAF V600E (threshold of 5%) by means of Fisher’s exact test, and also observed a significant difference between the classes (Fisher’s exact test, one-tailed: p = 0.013) (Table 2). To further analyze the relevance of this observation, we performed a simple predictive modelling based on logistic regression [15]. First, we balanced the two groups with regard to sex (p-value after two-sample t-test of p = 0.19) and age (p-value after two-sample t-test of p = 0.136). The size of the two balanced groups changed to n = 20 for the metastatic group and n = 16 for the non-metastatic one. The initial mean prediction capacity of the model was 0.65 (SD = 0.077). Prompted by this initial result, we decided to perform a more sophisticated machine learning approach. A power analysis, varying the effect size as a parameter, showed that for a power of 80% the corresponding effect size is: 0.97 which is really close to observed effect size in our classification (Cohen effect size measure based on mean differences, metastatic samples vs non-metastatic sample, = 0.95). Therefore, our sample size would be equivalent to that one in other studies requiring 80% of prediction power with the observed effect size. Given our sample size, one might wonder whether it is sufficient to correctly observe any effect while keeping the Type II error under control. This is usually accomplished at a certain significance level by running a power analysis, where a priori the expected effect size must be known. Here, and given the lack of previous studies reporting BRAF differences between Metastatic and Disease Free group conditions, we used our observed difference in the means between both groups as an estimation of the population effect size. At the standard 5% significance level, this assumption provides a decent power of approximately 80% in our dataset.

**Table 2:** Mutational load comparison between stage II patients groups.

	Mutational load of BRAF V600E (%)	
Prognosis	<5%	>5%
Metastasis	18	3
Disease-free	8	9

Therefore we performed a machine learning approach using a pruned Decision Tree classifier, implemented in ('scikit-learn' [16]), to predict the metastatic response (see Material and Methods). After performing ‘Leave-One-Out’ cross-validation, the classification accuracy was measured using different metrics, such as accuracy = 0.75, precision = 0.72 and recall = 0.9. The cut-off value of the classifier is 33.05, which means that patients with a mutational load of BRAF V600E lower than 33.05% are classified as “bad prognosis” and those with a value higher than 33.05% are classified as “good prognosis”. The difference can also be visualized in a Kaplan-Meier curve (S2 Fig).

Then, we tested if BRAF V600E load was a better predictor than the traditional Breslow thickness value. We considered three scenarios: 1. using only the Breslow thickness as a predictor variable, 2. only the variable mutational load of BRAF V600E, or 3. Combining the two. The classification metric scores for a Decision Tree classifier were better for BRAF V600E alone (accuracy = 0.75, precision = 0.72 and recall = 0.9) than Breslow Thickness alone (accuracy = 0.69, precision = 0.6 and recall = 0.8) or both variables together (accuracy = 0.72, precision = 0.69 and recall = 0.8). Therefore, adding the Breslow variable to BRAF V600E load into the classifier did not increase performance. The best classification was achieved when BRAF V600E load variable was the only predictor variable.

Based on our results, stage II patients with low mutational load of BRAF V600E seem to have a higher risk of developing metastasis. Even though we are aware of the modest sample size, as a preliminary approach, our results suggest that the BRAF V600E mutational load can constitute a useful biomarker that is worth exploring further collectively by the melanoma community.

BRAF V600E load has been previously analyzed in melanoma samples by dropled digital PCR (ddPCR) in order to compare its precision with other techniques [6, 17]. With this study we extent previous works and confirm that QuantStudio 3D Digital PCR System is able to accurately measure V600E load. Furthermore, to our knowledge, our work is the first one analyzing the relationship between BRAF V600E load and prognosis.

These results do not attempt to constitute evidence against the treatment with BRAF V600E inhibitors. Evidently, BRAF inhibitors selectively attack BRAF mutated cells. However, further research about BRAF V600E mutational load could help understand its implication in the prognosis.

## Conclusions

The great variability of BRAF V600E mutational load observed in the samples analyzed, highlights the fact that tumor heterogeneity is a common feature in melanoma, where several subclones can coexist, with different abilities and behaviors. Interestingly, our results suggest that BRAF V600E is a promising biomarker of prognosis in AJCC stage II melanoma patients, which may help to improve the personalized medical care and survival of melanoma patients.

## Supporting information

S1 Fig Selection of tumoral area from a melanoma biopsy. A representative HE stained image of a superficial spreading melanoma (A) and the selection of tumoral area on the same image (B). The area determined by the blue line corresponds to the tumoral area.
(TIF)

S2 Fig Kaplan-Meier curve for stage II patients, regarding disease prognosis. (TIF)

S1 Table BRAF V600E mutational load in 10 different commercial melanoma cell lines. (DOCX)

S2 Table BRAF V600E mutational load in stage II samples. (DOCX)

S3 Table Metrics for the Decision Tree classifiers made in the machine learning approach for different variables. (DOCX)

S4 Table Cox multivariate analysis. (DOCX)

S5 Table Cox multivariate analysis with the transformed BRAF variable, according to the cut-off value of the Decision Tree classifier (33.05%). (DOCX)

S1 Text Testing Digital PCR (dPCR) in commercial melanoma cell lines. (DOCX)

S2 Text Correcting for non-tumor cells. (DOCX)

S3 Text Comparison of the predicting capacity of BRAF V600E load with Breslow thickness and ulceration. (DOCX)

S4 Text Cox multivariate analysis. (DOCX)

## Abbreviations

PCR: Polymerase chain reaction
dPCR: Digital PCR
AJCC: American Joint Committee on Cancer
FFPE: Formalin fixed paraffin embedded
IHC: Immunohistochemistry
