# Supplementary figures and images for: BRAF V600E mutational load as a prognosis biomarker in malignant melanoma
Source: PLoS One. 2020 Mar 13;15(3):e0230136. doi: 10.1371/journal.pone.0230136 (PMC7069620; doi:10.1371/journal.pone.0230136)

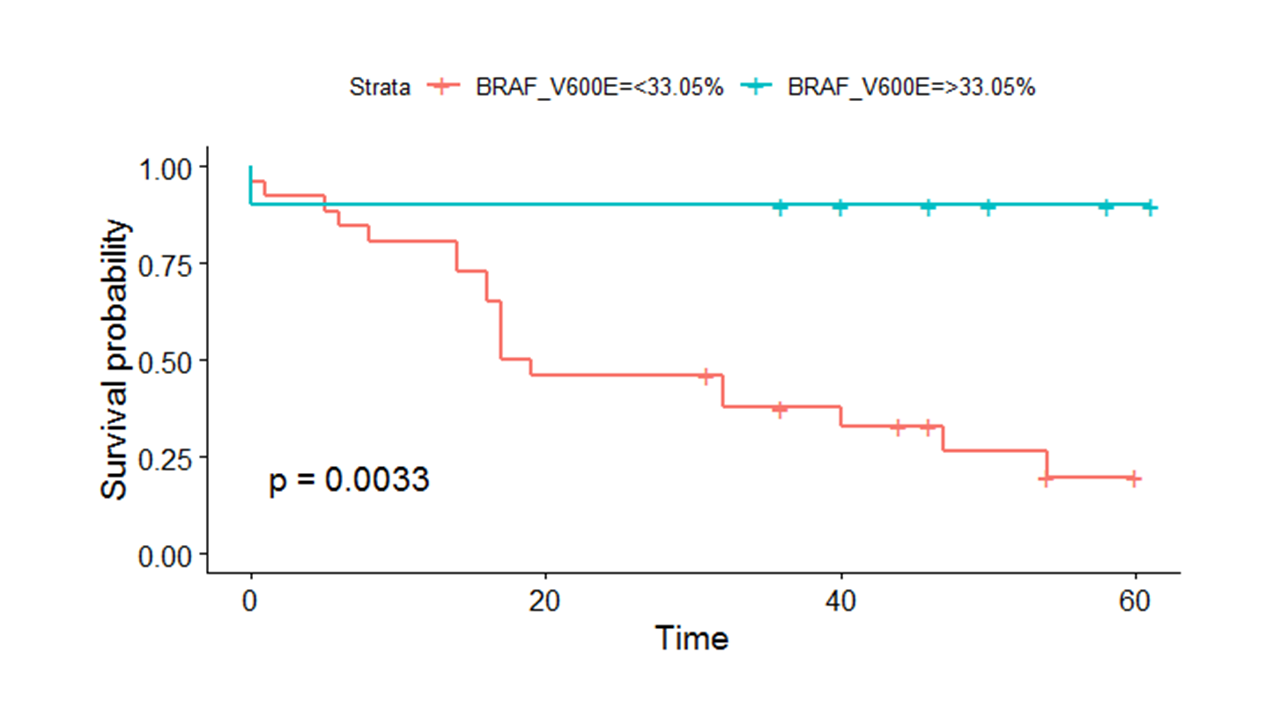

Supplement: S2 Fig — (TIF) [file pone.0230136.s002.tif]
