# Supplementary material for: BRAF V600E mutational load as a prognosis biomarker in malignant melanoma
Source: PLoS One. 2020 Mar 13;15(3):e0230136. doi: 10.1371/journal.pone.0230136 (PMC7069620; doi:10.1371/journal.pone.0230136)
Supplement: S1 Table — (DOCX) [file pone.0230136.s003.docx]

**S1 Table. BRAF V600E mutational load in 10 different commercial melanoma cell lines.**

| melanoma  cell line | origin | % BRAF V600E  (dPCR) |
| --- | --- | --- |
| RPMI 7951 | metastasis | 0 |
| WM-266-4 | metastasis | 0 |
| SKMEL-2 | metastasis | 0.01 |
| SKMEL-3 | metastasis | 19.09 |
| Hs-294t | metastasis | 54.6 |
| COLO-800 | metastasis | 70.52 |
| G361 | primary | 71.25 |
| A375 | primary | 100 |
| HT-144 | metastasis | 100 |
| SKMEL-28 | metastasis | 100 |
