# Supplementary material for: BRAF V600E mutational load as a prognosis biomarker in malignant melanoma
Source: PLoS One. 2020 Mar 13;15(3):e0230136. doi: 10.1371/journal.pone.0230136 (PMC7069620; doi:10.1371/journal.pone.0230136)
Supplement: S2 Table — (DOCX) [file pone.0230136.s004.docx]

**S2 Table.** BRAF V600E mutational load in stage II samples.

| Sample | Metastasis (YES/NO) | BRAF V600E load (%) |
| --- | --- | --- |
| 1 | NO | 1.12 |
| 2 | NO | 35.62 |
| 3 | NO | 0.00 |
| 4 | NO | 62.75 |
| 5 | NO | 46.39 |
| 6 | NO | 34.73 |
| 7 | NO | 39.15 |
| 8 | NO | 0.00 |
| 9 | NO | 0.00 |
| 10 | NO | 0.09 |
| 11 | NO | 0.05 |
| 12 | NO | 56.66 |
| 13 | NO | 57.82 |
| 14 | NO | 58.27 |
| 15 | NO | 0.00 |
| 16 | NO | 0.08 |
| 17 | NO | 40.13 |
| 18 | YES | 1.04 |
| 19 | YES | 0.00 |
| 20 | YES | 0.00 |
| 21 | YES | 0.00 |
| 22 | YES | 0.22 |
| 23 | YES | 31.37 |
| 24 | YES | 0.00 |
| 25 | YES | 0.02 |
| 26 | YES | 0.00 |
| 27 | YES | 0.00 |
| 28 | YES | 0.00 |
| 29 | YES | 10.53 |
| 30 | YES | 1.42 |
| 31 | YES | 0.00 |
| 32 | YES | 0.00 |
| 33 | YES | 1.62 |
| 34 | YES | 0.00 |
| 35 | YES | 0.00 |
| 36 | YES | 0.17 |
| 37 | YES | 0.00 |
| 38 | YES | 76.40 |
