# Supplementary material for: BRAF V600E mutational load as a prognosis biomarker in malignant melanoma
Source: PLoS One. 2020 Mar 13;15(3):e0230136. doi: 10.1371/journal.pone.0230136 (PMC7069620; doi:10.1371/journal.pone.0230136)
Supplement: S3 Table — (DOCX) [file pone.0230136.s005.docx]

**S3 Table.** Metrics for the Decision Tree Classifiers made in the machine learning approach for different variables.

| Variables | Metrics | | | |
| --- | --- | --- | --- | --- |
|  | **Accuracy** | **Recall** | **Precision** | **F1 score** |
| BRAF V600E load | **0.77** | **0.84** | **0.76** | **0.80** |
| Breslow Thickness | 0.71 | 0.80 | 0.63 | 0.72 |
| Ulceration | 0.60 | 0.73 | 0.60 | 0.66 |
| BRAF V600E load + Breslow Thickness | 0.68 | 0.78 | 0.68 | 0.73 |
| BRAF V600E load + Ulceration | **0.77** | **0.68** | **0.86** | **0.76** |
| Breslow Thickness + Ulceration | 0.71 | 0.80 | 0.63 | 0.70 |
| BRAF V600E load + Breslow Thickness + Ulceration | 0.60 | 0.68 | 0.61 | 0.65 |
