# Supplementary material for: BRAF V600E mutational load as a prognosis biomarker in malignant melanoma
Source: PLoS One. 2020 Mar 13;15(3):e0230136. doi: 10.1371/journal.pone.0230136 (PMC7069620; doi:10.1371/journal.pone.0230136)
Supplement: S4 Table — (DOCX) [file pone.0230136.s006.docx]

**S4 Table**. Cox multivariate analysis.

|  | coef | exp(coef) | se(coef) | z | p |
| --- | --- | --- | --- | --- | --- |
| BRAF_V600E | -0.0285 | 0.9719 | 0.0168 | -1.69 | 0.091 |
| Age | 0.0213 | 1.0215 | 0.0190 | 1.12 | 0.263 |
| GenderMale | -0.9385 | 0.3912 | 0.4804 | -1.95 | 0.051 |
| Ulceration | 0.2566 | 1.2925 | 0.4074 | 0.63 | 0.529 |
| Breslow | 0.3348 | 1.3977 | 0.1374 | 2.44 | 0.015 |
