# Supplementary material for: BRAF V600E mutational load as a prognosis biomarker in malignant melanoma
Source: PLoS One. 2020 Mar 13;15(3):e0230136. doi: 10.1371/journal.pone.0230136 (PMC7069620; doi:10.1371/journal.pone.0230136)
Supplement: S5 Table — (DOCX) [file pone.0230136.s007.docx]

**S5 Table**. Cox multivariate analysis with the transformed BRAF variable, according to the cut-off value of the Decision Tree Classifier (33.05%).

|  | coef | exp(coef) | se(coef) | z | p |
| --- | --- | --- | --- | --- | --- |
| BRAF_V600E  >33.05% | -2.1155 | 0.1206 | 1.0438 | -2.03 | 0.043 |
| Age | 0.0255 | 1.0259 | 0.0178 | 1.44 | 0.151 |
| GenderMale | -0.9268 | 0.3958 | 0.4793 | -1.93 | 0.053 |
| Ulceration | 0.2829 | 1.3269 | 0.4058 | 0.70 | 0.486 |
| Breslow | 0.3035 | 1.3545 | 0.1373 | 2.21 | 0.027 |
