# Supplementary material for: BRAF V600E mutational load as a prognosis biomarker in malignant melanoma
Source: PLoS One. 2020 Mar 13;15(3):e0230136. doi: 10.1371/journal.pone.0230136 (PMC7069620; doi:10.1371/journal.pone.0230136)
Supplement: S1 Text — (DOCX) [file pone.0230136.s008.docx]

**S1 Text. Testing Digital PCR (dPCR) in commercial melanoma cell lines**

We tested the ability of the BRAF V600E dPCR system to detect heterogeneity in 10 different commercial melanoma cell lines. The results are shown in Table S1 (below). The system can detect several loads from 0% to 100% of BRAF V600E.
