# Supplementary material for: BRAF V600E mutational load as a prognosis biomarker in malignant melanoma
Source: PLoS One. 2020 Mar 13;15(3):e0230136. doi: 10.1371/journal.pone.0230136 (PMC7069620; doi:10.1371/journal.pone.0230136)
Supplement: S2 Text — (DOCX) [file pone.0230136.s009.docx]

**S2 Text. Correcting for non-tumor cells**

In reporting the percentage of the BRAF V600E allele (BRAF V600E mutational load) for each biopsy slice, two possible sources of noise were taken into consideration:

a) The proportion of surrounding healthy tissue

To remove the contribution of DNA from the surrounding healthy tissue in the dPCR analysis, hematoxylin-eosin (HE) stained, scanned images of each biopsy were visually inspected to estimate the proportion of tumour-only area (TOA) (see Figure S1) vs the total biopsy area (TBA). Tumoral areas were measured using ImageJ software. Then, assuming that mutated BRAF counts could only come from the tumor tissue, and not from the surrounding healthy tissue, the estimate of the corrected number of wild type alleles (CWT) in each biopsy was:

CWT=(uncorrected #wt alleles)*(TOA/TBA)

and then the corrected load is

100 X (#mutant alleles)/(corrected #wt alleles + #mutant alleles)

We have attempted to compare our results with those from the TCGA collection. However, comparison is difficult, as the estimation of mutant alleles reported in that case was done by counting the number of reads (obtained by NGS) at a median depth of ~100X (in contrast, our median number of PCR reactions were ~1000). At that depth, reliable estimation of mutant allele load may not be feasible for loads, say, ~<10-20% (see for instance Carter et *al*. (2012) Absolute quantification of somatic DNA alterations in human cancer. Nat Biotechnol. 30:413–421. Portier et *al*. (2014) Quantitative Assessment of Mutant Allele Burden in Solid Tumors by Semiconductor-Based Next-Generation Sequencing. Am J Clin Pathol 141:559-572). On the other hand, TCGA samples include both primary and metastatic tumors, whereas in our case only primary tumors were analyzed.

b) After correcting for this potential source of bias, however, it is still possible to argue that tumors contain infiltrating non-tumoral cells that could also be contributing unaccountedly to the fraction of wild-type BRAF detected, thereby decreasing the relative proportion of BRAF V600E mutational load. We believe that this is not a strong interfering factor, because on the one hand, it has been estimated that in the skin melanomas from TCGA, the median proportion of tumor-infiltrating lymphocytes (TILs) is less than 5% (Saltz et *al*. 2018. Spatial Organization and Molecular Correlation of Tumor-Infiltrating Lymphocytes Using Deep Learning on Pathology Images. Cell Reports 23:181–193). On the other hand, we consider the quantification of TILs irrelevant for the purpose of this manuscript, as what we are reporting as a prognosis factor is an estimation of the mutational load per unit of tumoral area. Whether this varies according to the proportion of TIL cells (or other) within the tumoral tissue or according to the variation in the number of mutated alleles (within the melanocytes of the tumoral tissue) is not the target of this paper (although it would be interesting to know). Whatever the reason, the score we provide shows a correlation with prognosis in stage II melanomas (higher BRAF V600E load, better prognosis).

Finally, we are no attempting in this paper to offer a biological explanation why this is so. However, we can speculate that, perhaps, a higher proportion of mutated BRAF elicits a stronger immune response against melanoma-associated antigens in patients (Ileva et *al*. 2014. Effects of BRAF Mutations and BRAF Inhibition on Immune Responses to Melanoma. Mol Cancer Ther.13:2769–83) that could result in a better prognosis (in this case we can only refer to stage II patients).
