# Supplementary material for: BRAF V600E mutational load as a prognosis biomarker in malignant melanoma
Source: PLoS One. 2020 Mar 13;15(3):e0230136. doi: 10.1371/journal.pone.0230136 (PMC7069620; doi:10.1371/journal.pone.0230136)
Supplement: S3 Text — (DOCX) [file pone.0230136.s010.docx]

**S3 Text. Comparison of the predicting capacity of BRAF V600E load with Breslow Thickness and Ulceration.**

We performed the previously used machine learning approach (Decision Tree Classifier) adding the Ulceration variable. We performed the analysis with only 35 samples for which we did have information for Ulceration. We tested BRAF V600E mutational load alone, Breslow alone, Ulceration alone and combinations of all the variables and the best results are still for BRAF V600E load alone (S3 Table). However, BRAF V600E load and Ulceration variables together also show good results. The accuracy is the same as using BRAF V600E load alone. However, the recall is higher for BRAF V600E load and the precision is higher for both variables together. For the purpose of the analysis, we though that having a higher recall is more desirable. In this case, the positive cases (metastatic patients) would be better classified. Besides, looking at F1 metric, which summarizes the precision and recall, BRAF V600E alone has a higher value.
