# Supplementary material for: BRAF V600E mutational load as a prognosis biomarker in malignant melanoma
Source: PLoS One. 2020 Mar 13;15(3):e0230136. doi: 10.1371/journal.pone.0230136 (PMC7069620; doi:10.1371/journal.pone.0230136)
Supplement: S4 Text — (DOCX) [file pone.0230136.s011.docx]

**S4 Text. Cox multivariate analysis.**

We have also performed a Cox multivariate analysis. We have consider the variables BRAF V600E load, Breslow Thickness, Ulceration, age and gender. The test is not significant for BRAF V600E variable (see S4 Table). However, when we transform the variable according to the cut-off value of the Decision Tree Classifier (33.05%), it appears significant (p=0.043) (see S5 Table).

Nevertheless, Cox analysis is a semiparametric model that assumes a lineal relationship between the independent variables with respects to the hazard function. A lineal relationship could not model properly the behaviour of BRAF variable. For that reason, to perform the classification analysis, we have chosen a Decision Tree Classifier, which is a non-parametric method.
